# The clinical effect of sevoflurane anesthesia with laryngeal mask airway in the extraction of teeth in children

**DOI:** 10.3389/fped.2024.1415440

**Published:** 2024-07-25

**Authors:** Bin Wang, Minglin Han

**Affiliations:** ^1^ Department of Oral and Maxillofacial Surgery, Wuxi Stomatology Hospital, Wuxi, China; ^2^ Department of Preventive Dentistry, Wuxi Stomatology Hospital, Wuxi, China

**Keywords:** sevoflurane, anesthesia for children, laryngeal mask airway, extraction of teeth, postoperative recovery

## Abstract

**Objective:**

To evaluate the effect of sevoflurane general anesthesia with laryngeal mask airway in the extraction of teeth.

**Methods:**

A retrospective analysis was performed on 88 children who underwent extraction of teeth in the Department of Anesthesiology of our hospital from June 2022 to April 2023, including 44 patients who received traditional anesthesia as the control group and 44 patients who received laryngeal mask airway sevoflurane general anesthesia as the observation group. Anesthesia and operation records of patients in the two groups were analyzed, including intraoperative vital signs, anesthesia induction time, recovery time of spontaneous breathing, first feeding time within 24 h after surgery, postoperative pain score, incidence of adverse reactions, Ramsay score and wake agitation, and other indicators were collected, and statistical analysis was conducted.

**Results:**

The recovery time of the observation group was 7.88 ± 4.95 min, and the recovery time of spontaneous respiration was 10.58 ± 3.64 min, which were significantly shorter than 15.23 ± 5.12 min and 14.41 ± 3.56 min of the control group (*P < *0.001). There were no significant differences between the two groups in anesthesia induction, operation duration and first feeding time within 24 h after operation (*P > *0.05). There was no significant difference in postoperative pain scores between the two groups (*P > *0.05). The overall incidence of adverse reactions was 6.82% in the observation group compared with 22.73% in the control group (*χ*² = 4.423, *P =* 0.035). In addition, the Ramsay score of the observation group was significantly improved compared with the control group (*P < *0.05), and the incidence of agitation during the recovery period was also significantly decreased (*P < *0.05).

**Conclusion:**

Laryngeal mask airway sevoflurane anesthesia can significantly accelerate the recovery process of children after extraction of teeth, and reduce the occurrence of adverse reactions, providing a safer and more efficient choice than traditional anesthesia.

## 1 Introduction

Pediatric oral surgery, especially the removal of ambushed overborn teeth, is a common oral surgery procedure, and anesthesia management becomes a crucial task in order to ensure the smooth operation and minimize the discomfort of the child (1). In recent years, with the development of anesthesiology and the emergence of new anesthetic drugs, the choice of anesthetics has become diversified. Among them, sevoflurane, as an inhaled anesthetic with rapid onset and recovery, has attracted more and more attention for its safety and comfort in pediatric patients. Sevoflurane can not only provide a stable anesthetic state, but also shorten the postoperative recovery time and reduce the risk of postoperative aspiration and vomiting and other adverse reactions, which is conducive to improving the anesthesia experience of children patients (2, 3).

However, compared with adults, pediatric patients have different physiological characteristics and drug metabolism mechanisms, and how to optimize pediatric anesthesia management to obtain the best clinical results is still one of the challenges facing anesthesiology research (4). In addition, children's oral surgery is often performed in a confined space, requiring more delicate anesthesia operations, especially to maintain airway patency is crucial for the success of the operation (5). In this context, laryngeal mask airway, as an ideal airway management method, has been widely used in pediatric anesthesia due to its advantages such as simplicity of operation, little irritation to the respiratory tract and easy fixation (6, 7).

Hence, the primary objective of this retrospective study is to evaluate the efficacy of sevoflurane general anesthesia with laryngeal mask airway in the extraction of teeth in children. Secondary objectives are to compare intraoperative vital signs stability between the two anesthesia methods, assess postoperative pain control using visual analogue scale (VAS) scores, measure the time required for the child to wake up and move from the operating room to the recovery room, evaluate the incidence of postoperative nausea, vomiting, and other adverse reactions, and analyze the Ramsay score and incidence of postoperative agitation during the recovery period. The comparison was performed between the control group that received traditional anesthesia and the observation group receiving laryngeal mask airway with sevoflurane laryngeal mask airway.

## 2 Data and methods

### 2.1 Study design and participants

This retrospective study included children who underwent extraction of teeth in our Department of Anesthesiology between June 2022 and April 2023. After rigorous screening, a total of 88 children were included, ranging in age from 4 to 12 years. The American Society of Anesthesiology (ASA) classification was I or II, and there was no significant dysfunction of vital organs such as heart, liver and kidney, which met the conditions of general anesthesia. The study was approved on 19th March, 2023, by the Ethics Review Committee (approval number: HEC2022-058) in accordance with the ethical statement of the Declaration of Helsinki.

Inclusion criteria: children between the ages of 4 and 12; Diagnosed as ambushed redundant teeth and planned for extraction; Body rating ASA I or II; The legal guardian has signed the informed consent.

Exclusion criteria: A history of severe allergy to known sevoflurane or other inhaled narcotic drugs; Have serious cardiovascular, respiratory or central nervous system disease and are not suitable for general anesthesia; Use of analgesics or other drugs that affect the effect of anesthesia within 48 h prior to surgery; Has inherited myopathy or other diseases that could increase the risk of intraoperative complications; Other conditions deemed inappropriate for inclusion in the study by the surgeon or anesthesiologist.

### 2.2 Methods

This retrospective study was divided into two groups according to the method of anesthesia: (1) control group and (2) observation group. Before the surgery, the anesthesiologist had sufficient communication with the children and their parents to alleviate the concerns of the children about the surgical process. Fasting began 6 h before surgery, and drinking was prohibited 2 h before surgery. After entering the operating room, the patient received a slow intravenous infusion of propofol of 2 mg/kg through an upper limb venous channel to achieve sedation. During this period, the child was given oxygen through a mask and the oxygen flow was maintained at 6 L/min.

Control group: After the assured basic vital signs were stable, the patients received anesthesia induction with the following drugs: dexamethasone 0.2 mg/kg, midazolam 0.04 mg/kg, cisatracurium 0.08 mg/kg, and then sufentanil 0.5 ug/kg to provide intraoperative analgesia. With medication, the child was intubated and connected to a ventilator. The depth of anesthesia and respiratory parameters were adjusted according to the physiological response of the child.

Observation group: After basic sedation, the children in this group received the same dose of propofol, dexamethasone, midazolam and cisatracurium as the control group. Then sevoflurane inhalation anesthesia was performed by laryngeal mask airway. The initial induction concentration was set at 8%, the maintenance concentration was adjusted to 2%–3%, and the oxygen flow was reduced from the initial 6 L/min to 2 L/min to maintain stable anesthesia during the operation. Propofol infusion was initiated for sedation and anesthesia induction but was discontinued upon the transition to sevoflurane inhalation anesthesia via laryngeal mask airway.

After achieving the desired level of anesthesia, both groups of children underwent standard surgical procedures performed by oral surgeons. After mucosal incision, appropriate surgical instruments were used to remove the resistance around the alveolar bone, and then the embedded tooth was carefully removed. After the operation, the wound was sutured with absorbable thread. All children underwent rigorous vital signs monitoring during surgery and anesthesia, including electrocardiogram, oxygen saturation, noninvasive blood pressure, and end-tidal carbon dioxide concentration. The use of laryngeal mask airway in the observation group provided a secure airway seal, reducing the likelihood of aspiration. Additionally, careful suctioning of the oral cavity was performed throughout the procedure to remove excess fluids. Surgeons were trained to minimize the presence of fluids near the airway and to promptly address any potential airway contamination. After surgery, the child was transferred to the resuscitation room until safe recovery, and transferred to the ward for continued monitoring after meeting the exit criteria.

Intraoperatively, the primary analgesic administered was sufentanil at a dose of 0.5 ug/kg to ensure adequate pain control. Postoperatively, analgesia management included the use of paracetamol (acetaminophen) as a standard protocol for pain relief.

### 2.3 Outcome measures

The outcome measures included intraoperative vital sign stability, i.e., fluctuation of heart rate, blood pressure, oxygen saturation, and respiratory rate throughout the procedure. In addition, pain management is a core concern, and postoperative pain scores will be quantified using a visual analogue scale (VAS) to assess differences in pain control between the two groups. The time to induction of anesthesia, the time for the child to wake up, and the time required for the child to move from the operating room to the recovery room were calculated to measure the speed of recovery. Nausea, vomiting and other possible adverse reactions during postoperative recovery were also recorded and their impact on the quality of recovery was evaluated. Finally, Ramsay score and postoperative agitation were investigated and recorded in all children.

All children in this study were admitted postoperatively to a pediatric ward for monitoring and management. The duration of admission depended on individual recovery progress and meeting discharge criteria. For those scheduled as day-case surgeries, we contacted parents within 24 h postoperatively to assess pain scores and monitor for any postoperative complications. Patient assessment and communication with parents postoperatively are integral parts of our management protocol, aimed at ensuring comprehensive recovery and effective pain management.

### 2.4 Statistical analysis

With a moderate effect size of 0.5, a two-group independent samples *t*-test design would require a total sample size of at least 70 participants (35 per group) to detect a significant difference between the treatment and control groups. However, to account for potential dropouts or missing data, the sample size was increased to 88 participants.

Before statistical analysis, an α value of 0.05 was set to determine the threshold of statistical significance. If the continuous variables are normally distributed, *t*-test is used to analyze the difference between the two groups. Continuous variables with non-normal distributions are tested by Man-Whitney *U*-test. The class variables were analyzed by Chi-square test. All statistical analyses were performed using SPSS statistical software.

## 3 Results

This retrospective study evaluated the clinical effect of sevoflurane anesthesia with laryngeal mask airway in the extraction of teeth. The results included baseline characteristics, intraoperative vital signs data, time to anesthesia and postoperative recovery, postoperative pain score, incidence of adverse reactions, Ramsay score, and emergence and agitation.

### 3.1 Baseline characteristics

Table 1 shows a comparison of baseline characteristics between the two groups, including age, weight, body mass index (BMI), length of surgery, and ASA scores. There were no significant differences in these variables between the two groups (*P > *0.05), indicating that the data of the two groups were comparable.

**Table 1:** Comparison of characteristics of children at baseline.

	Age (years)	Sex (male/female)	Weight (kg)	Body mass index (kg/m^2^)	Duration of operation (minutes)	American society of anesthesiology classification (I/II)
Control group (*n* = 44)	8.5 ± 2.1	20/24	27.4 ± 8.5	17.5 ± 2.9	90.5 ± 25.1	20/22
Observation group (*n* = 44)	8.5 ± 2.1	25/19	26.8 ± 8.8	18.0 ± 3.2	88.5 ± 24.8	22/22

### 3.2 Intraoperative vital signs

**Figure 1:**
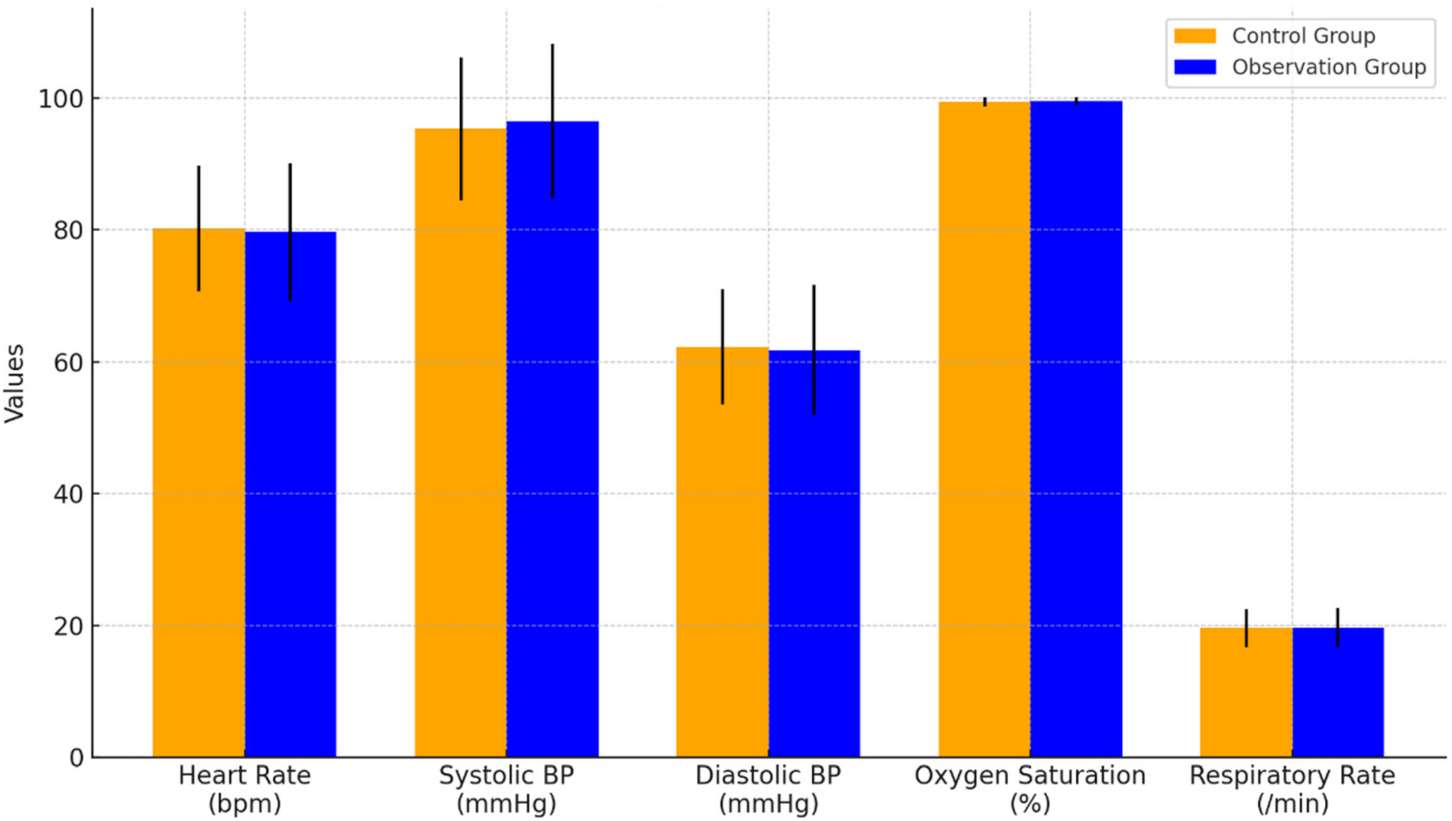
Intraoperative vital signs data.

Figure 1 shows the vital signs data of the two groups of children during surgery, including heart rate, systolic blood pressure, diastolic blood pressure, oxygen saturation, and respiratory rate. The results showed that there was no significant difference between the two groups (*P > *0.05).

### 3.3 Time related to anesthesia and postoperative recovery

Table 2 presents data on the time associated with anesthesia and postoperative recovery. The results showed that although there were no significant differences in anesthesia induction time, operation time and first postoperative feeding time between the two groups, the recovery time and spontaneous breathing recovery time in the observation group were significantly shorter than those in the control group, with statistical significance (*P < *0.001).

**Table 2:** Comparison of anesthesia and recovery parameters between control and observation groups.

Group	Anesthesia induction time (min)	Surgery duration (min)	Recovery time (min)	Autonomous breathing recovery time (min)	First feeding time post-op (min)
Control group (*n* = 44)	13.5 ± 1.3	88.7 ± 22.1	15.9 ± 2.4	16.6 ± 2.2	252.8 ± 62.1
Observation group (*n* = 44)	14.0 ± 1.2	86.5 ± 20.4	10.5 ± 1.7	13.9 ± 1.4	250.3 ± 60.8
*t*	−1.88	0.43	10.75	9.55	0.12
*P*	0.064	0.671	0.000	0.000	0.907

### 3.4 Postoperative pain score

**Figure 2:**
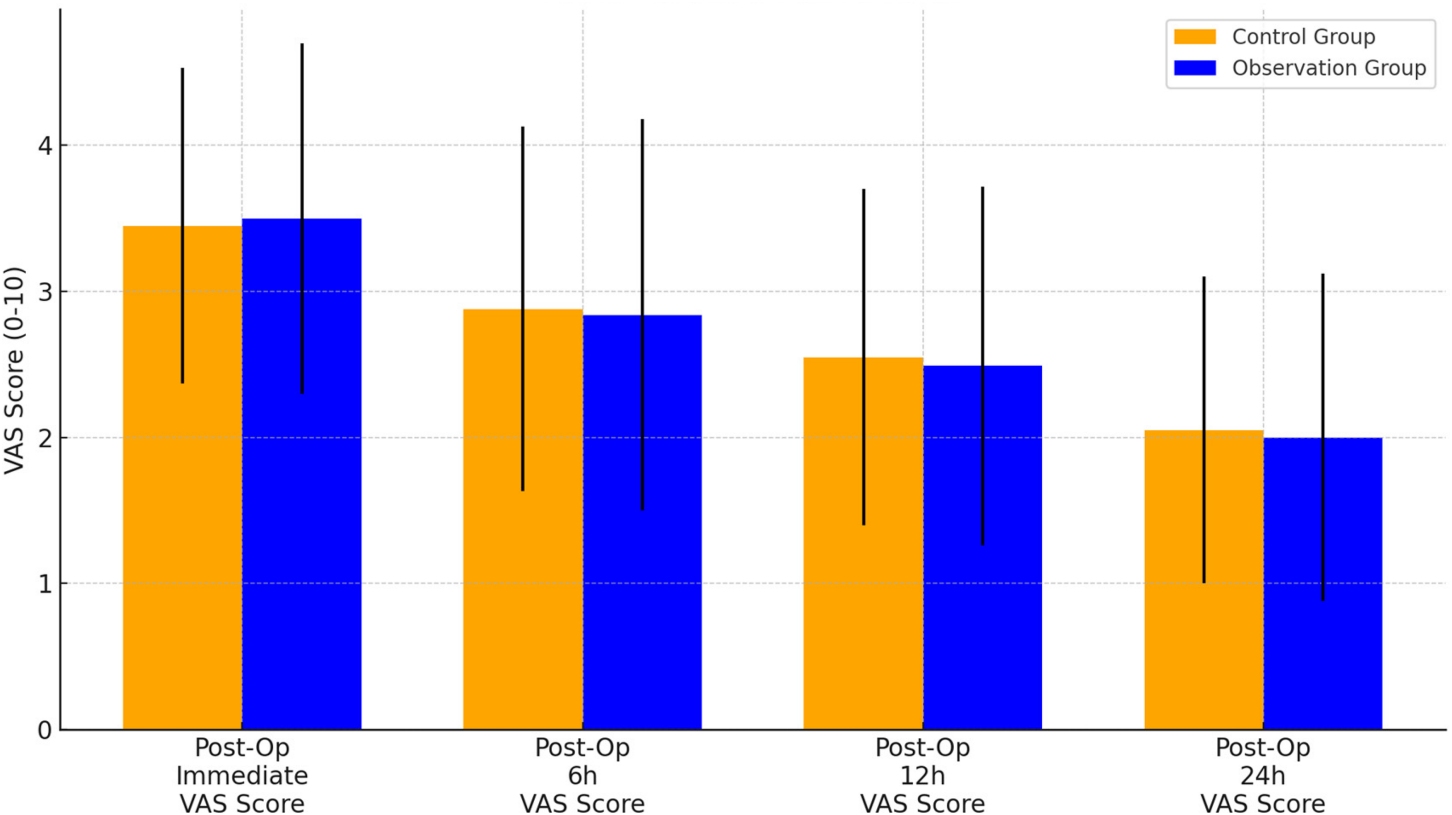
Postoperative pain score. VAS, visual analogue scale.

Figure 2 shows the results of VAS pain scores immediately, 6 h, 12 h, and 24 h after surgery in the two groups. There were no significant differences in VAS scores between the two groups at these 4 time points (*P > *0.05).

### 3.5 Comparison of the incidence of adverse reactions

There were differences in the incidence of nausea, vomiting, respiratory depression and emotional irritability between the observation group and the control group. Among the 44 children in the control group, adverse reactions included nausea in 4 cases (9.09%), vomiting in 1 case (2.27%), respiratory depression in 2 cases (4.55%), and emotional irritability in 3 cases (6.82%), totaling 10 cases (22.73%) with adverse reactions. In contrast, among the 44 children in the observation group, 2 cases (4.55%) reported nausea, no cases of vomiting or respiratory depression were observed, and 1 case (2.27%) reported emotional irritability, resulting in a total of 3 cases (6.82%) with adverse reactions. The comparison of adverse reactions between the two groups showed a significant difference (*χ*² = 4.423, *P =* 0.035), as shown in Table 3.

**Table 3:** Comparison of adverse reactions between the two groups [*n* (%)].

	Nausea	Vomit	Respiratory depression	Emotional irritability	Total
Control group (*n* = 44)	4 (9.09%)	1 (2.27%)	2 (4.55%)	3 (6.82%)	10 (22.73%)
Observation group (*n* = 44)	2 (4.55%)	0 (0.00%)	0 (0.00%)	1 (2.27%)	3 (6.82%)
*χ²*	0.668	–	–	0.262	4.423
*P*	0.414	1.000	1.000	0.609	0.035

### 3.6 Comparison of Ramsay scores

The Ramsay score was similar between the observation group and the control group. At 1 h after operation, the scores of the observation group and the control group were reduced to 2.96 ± 0.34 and 2.55 ± 0.21 respectively, and the scores of the observation group were higher than those of the control group (*P < *0.001). At 3 h after surgery, the Ramsay score difference between the two groups was greater, with the observation group being 2.21 ± 0.23 and the control group 1.79 ± 0.15, and the difference between the two groups was statistically significant (*P < *0.001), as shown in Table 4.

**Table 4:** Comparison of postoperative Ramsay scores between the two groups.

	Awakening	1 h after surgery	3 h after surgery
Control group (*n* = 44)	4.15 ± 0.33	2.55 ± 0.21	1.79 ± 0.15
Observation group (*n* = 44)	4.29 ± 0.42	2.96 ± 0.34	2.21 ± 0.23
*t*	−1.739	−6.805	−10.146
*P*	0.086	0.000	0.000

### 3.7 Comparison of postoperative agitation

The comparison of postoperative recovery and agitation of the two groups was shown in Table 5. Post Anesthetic Emergence Delirium (PAED) score of the observation group was 13.22 ± 1.86, and that of the control group was 8.93 ± 1.54, which was significantly lower in the observation group than in the control group (*P < *0.001). In terms of the number and proportion of agitation, the observation group was significantly lower than the control group (*P < *0.05).

**Table 5:** Comparison of postoperative recovery and agitation between the two groups.

	PAED score	Emergence agitation [*n* (%)]
Control group (*n* = 44)	13.22 ± 1.86	18 (40.91%)
Observation group (*n* = 44)	8.93 ± 1.54	8 (18.18%)
*t/χ* ^2^	11.784	5.459
*P*	0.000	0.019

PAED, post anesthetic emergence delirium.

## 4 Discussion

When children undergo oral surgery, their faces are often covered with a hole towel, which often causes them tension. Considering that these patients are still young, their psychological development is not mature, and they have a strong dependence on their parents, they often cannot express their needs clearly, and usually cry to express discomfort (8). As a result, dealing with children's oral health is challenging in clinical practice. Due to the active nature of children, it is difficult to follow medical advice, which may lead to the delay of disease treatment (9). Traditional oral treatment for children mostly uses compression, which causes many young patients to finish the treatment while crying, which not only affects the recovery process of the disease, but also may cause conflicts between doctors and patients (10). With the improvement of medical technology and the increase of people's demand for medical comfort, the application of anesthesia technology in children's oral therapy is becoming more and more extensive. General anesthesia drugs, as a class of drugs that can temporarily inhibit the function of the central nervous system, can temporarily inactivate the consciousness, perception and reflexes of patients, while relaxing the skeletal muscle (11, 12).

The opioid component found in opioids offers notable pain-relieving, cough-suppressing, anti-diarrheal, sedative, and sleep-inducing effects. However, it is important to note that it can also lead to certain adverse reactions, including alterations in consciousness, drowsiness, feelings of nausea, vomiting, and difficulties with urinary function. Sufentanil is a powerful analgesic, which has a strong binding ability with opioid receptors, and its analgesic effect is more significant and the action time is longer (13). In the liver, sufentanil undergoes extensive biotransformation to produce metabolites of n-dehydroxy and o-demethylation, which are excreted through the kidneys. Sevoflurane is commonly used as a general anesthetic and is favored for its low tracheal irritation, smooth and rapid anesthesia guidance and wakefulness, and easy adjustment of the depth of anesthesia. In addition, sevoflurane has a short induction time and high safety (14, 15).

The purpose of this study was to evaluate the clinical effect of sevoflurane anesthesia with laryngeal mask airway in the extraction of teeth in children. The results show that in clinical practice, the use of sevoflurane as an inhalation anesthetic program has certain advantages in improving postoperative recovery and reducing adverse reactions than the traditional program.

Combined with the study results, the observation group showed a faster time to wake up and recovery time with spontaneous breathing. Specifically, children in the control group woke up in an average of 15.23 min, compared with 7.88 min in the observation group. The recovery time of spontaneous respiration was 14.41 min in the control group and 10.58 min in the observation group. The recovery rate of the observation group was significantly better than that of the control group. This suggests that the use of the inhalational sevoflurane during general anesthesia can accelerate the induction and waking process of anesthesia. The rapid onset and recovery times of sevoflurane are primarily attributed to its pharmacokinetic properties rather than its hemodynamic effects. Sevoflurane has minimal impact on blood pressure upon inhalation, and its effects dissipate quickly after cessation, contributing to shorter patient recovery times. This finding is consistent with Naveen et al. (16), who observed in one study that patients using sevoflurane showed a tendency to recover more quickly after surgery compared to other inhaled anesthetics.

In terms of postoperative pain management, VAS scores were not significantly different between the two groups immediately after surgery, at 6 h, 12 h, and 24 h, indicating that sevoflurane did not show a significant advantage in pain control. This is consistent with the findings of Pentilas et al. (17). The incidence of adverse reactions was also significantly lower in the observation group than in the control group. Specifically, 22.73% of children in the control group experienced adverse reactions, compared to 6.82% in the observation group. Especially in the two common postoperative complications of nausea and vomiting, only 4.55% of the children in the observation group had nausea, and there was no vomiting or respiratory depression. This suggests that using general anesthesia with appropriate opioids may be safer for the extraction of teeth. Sufentanil is mainly converted in the liver and then excreted through the liver, so there are more side effects. These drugs should be used with greater caution in pediatric patients with liver or kidney insufficiency. Finally, the analysis of Ramsay scores in both groups demonstrated that the observation group achieved a significantly higher score in comparison to the control group. Furthermore, there was a notable reduction in the incidence of agitation during the recovery period in the observation group, indicating significant improvement.

This study has several limitations that should be acknowledged. The retrospective design introduces potential biases in data collection and analysis, and the single-center setting may limit the generalizability of the findings to other contexts. Additionally, the relatively small sample size restricts the statistical power, potentially affecting the robustness of the results. The focus on the immediate postoperative period does not account for long-term outcomes or complications associated with sevoflurane anesthesia with laryngeal mask airway. Furthermore, the study did not control for potential confounding factors such as surgeons' experience, specific surgical techniques, or variations in intraoperative management, which could influence the outcomes. One of the limitations of this study is the absence of blinding, and there was no implementation of measures to prevent participants, researchers, or outcome assessors from knowing the group assignments. This lack of blinding introduces the possibility of bias in the study results. Lastly, the study did not explore the impact of patient characteristics like age, gender, or comorbid conditions, which could affect the results. Future research should include larger, multi-center samples, long-term follow-up, and better control of confounding factors to confirm these findings and enhance their applicability.

## 5 Conclusion

In summary, our study indicates that compared to the control group maintained with propofol infusion and endotracheal intubation, the observation group using sevoflurane maintenance with a laryngeal mask airway showed certain advantages in children undergoing extraction of impacted deciduous teeth. However, we acknowledge the limitations due to the small sample size and the presence of multiple confounding factors in the study design. Further large-scale, rigorously controlled randomized trials are warranted to confirm these findings and to further evaluate the safety and efficacy of different anesthesia approaches in pediatric surgical settings.

## Data Availability

The original contributions presented in the study are included in the article/Supplementary Material, further inquiries can be directed to the corresponding author.
